# Population genomics of the killer whale indicates ecotype evolution in sympatry involving both selection and drift

**DOI:** 10.1111/mec.12929

**Published:** 2014-10-12

**Authors:** Andre E Moura, John G Kenny, Roy Chaudhuri, Margaret A Hughes, Andreanna J Welch, Ryan R Reisinger, P J Nico de Bruyn, Marilyn E Dahlheim, Neil Hall, A Rus Hoelzel

**Affiliations:** * School of Biological and Biomedical Sciences, Durham UniversitySouth Road, Durham, DH1 3LE, UK; † Department of Functional and Comparative Genomics, Institute of Integrative Biology, University of LiverpoolCrown Street, Liverpool, L69 7ZB, UK; ‡ Mammal Research Institute, Department of Zoology and Entomology, University of PretoriaPrivate Bag X20, Hatfield 0028, Pretoria, South Africa; § National Marine Mammal Laboratory, National Marine Fisheries Service7600 Sand Point Way N.E., Seattle, WA, 98115, USA

**Keywords:** adaptation, Cetacea, ecological genetics, population genomics, sympatric divergence

## Abstract

The evolution of diversity in the marine ecosystem is poorly understood, given the relatively high potential for connectivity, especially for highly mobile species such as whales and dolphins. The killer whale (*Orcinus orca*) has a worldwide distribution, and individual social groups travel over a wide geographic range. Even so, regional populations have been shown to be genetically differentiated, including among different foraging specialists (ecotypes) in sympatry. Given the strong matrifocal social structure of this species together with strong resource specializations, understanding the process of differentiation will require an understanding of the relative importance of both genetic drift and local adaptation. Here we provide a high-resolution analysis based on nuclear single-nucleotide polymorphic markers and inference about differentiation at both neutral loci and those potentially under selection. We find that all population comparisons, within or among foraging ecotypes, show significant differentiation, including populations in parapatry and sympatry. Loci putatively under selection show a different pattern of structure compared to neutral loci and are associated with gene ontology terms reflecting physiologically relevant functions (e.g. related to digestion). The pattern of differentiation for one ecotype in the North Pacific suggests local adaptation and shows some fixed differences among sympatric ecotypes. We suggest that differential habitat use and resource specializations have promoted sufficient isolation to allow differential evolution at neutral and functional loci, but that the process is recent and dependent on both selection and drift.

## Introduction

The scarcity of strong geographical barriers and the large dispersal abilities of many marine species have led to suggestions that selection is a dominant mechanism driving population differentiation (Naciri *et al.* 1999; Bierne *et al.* 2003). This view has been supported by studies showing genetic structure associated with environmental and physiological differences (Ruzzante *et al.* 1996; Beheregaray & Sunnucks 2001; Jorgensen *et al.* 2005; Palumbi & Lessios 2005; Atarhouch *et al.* 2007; White *et al.* 2010; Bowen *et al.* 2013). However, several examples are known among marine taxa where small-scale population differentiation has been attributed to complex phylogeographic scenarios involving differentiation in isolation due to cyclical climate change (Alvarado Bremer *et al.* 2005; Domingues *et al.* 2007, 2008), vicariance or the opening of dispersal corridors (Tringali 1999; de Bruyn *et al.* 2005; Puckridge *et al.* 2012).

Cetaceans have large dispersal abilities and long-life expectancies, and yet often show genetic differentiation over relatively small geographic scales (Hoelzel 2009). Some studies have suggested that small-scale population differentiation in these species is caused by adaptation to local environmental differences (Yoshida *et al.* 2001; Möller *et al*. 2010; Mendez *et al.* 2011; Amaral *et al.* 2012), although the inference is indirect. It is also the case that closely related species with very similar distribution patterns may show very different patterns of genetic differentiation, such as the fine-scale differentiation seen for bottlenose dolphins (*Tursiops truncatus*) in European waters (Natoli *et al.* 2005) compared to the lack of structure for common dolphins (*Delphinus delphis*) over the same range (Moura *et al.* 2013a). This may emphasize the importance of species-specific resource requirements and specializations towards the evolution of habitat dependence, philopatry and population differentiation (Yurk *et al.* 2002; Natoli *et al.* 2006; Hoelzel *et al.* 2007; Moura *et al.* 2013a).

Killer whales show population genetic structure over spatial scales that are much smaller than their dispersal abilities (Hoelzel *et al.* 2007). These large-bodied dolphins are distributed worldwide and organized into stable, matrifocal social groups called pods. Different communities of pods exhibit consistent, long-term specializations on prey resource, defining different ‘ecotypes’ (which sometimes also differ with respect to other aspects of behaviour and morphology, see de Bruyn *et al.* 2013). Although the level of gene flow between pods varies depending on the ecotype, gene flow between different ecotypes has been shown to be limited based on inference from both mtDNA and microsatellite DNA markers, with some exceptions (see Hoelzel *et al.* 2007; Morin *et al.* 2010; Foote *et al.* 2013). In the North Pacific, two ecotypes, known as ‘residents’ and ‘transients’, occupy largely sympatric distribution ranges (Ford *et al.* 2000), but are genetically well differentiated (e.g. Hoelzel *et al.* 2007). This is coincident with differences in prey specialization (fish vs. marine mammals, respectively, Ford *et al.* 1998; Krahn *et al.* 2007), social organization (Ford *et al.* 2000), mating systems (Pilot *et al.* 2010) and vocal behaviour (Yurk *et al.* 2002; Deecke *et al.* 2005).

Significant genetic differentiation is typically found for all comparisons of killer whale populations defined a priori either geographically or by ecotype (Hoelzel *et al.* 2007; Morin *et al.* 2010), although overall diversity is low worldwide, likely due to a bottleneck during the last glacial period (Hoelzel *et al.* 2002; Moura *et al.* 2014a). Differentiation is seen both between ecotypes in sympatry and following a pattern of isolation by distance within an ecotype (Hoelzel *et al.* 2007). However, previous studies restricted to neutral markers can provide only limited insight into the mechanisms of ecological adaptation and differentiation between ecotypes. Here we focus on the North Pacific, but include outgroup populations from the North Atlantic (Iceland) and Southern Oceans (Marion Island, MI). We use restriction-site-associated DNA (RAD) single-nucleotide polymorphic (SNP) markers to provide a high-resolution genomewide assessment of population structure at both neutral loci and markers putatively under selection. We test the hypothesis that populations representing sympatric ecotypes (e.g. residents and transients) will show patterns of differentiation that reflect selection at functional loci. More broadly, we investigate the hypothesis that in addition to the process of genetic drift, disruptive selection is driving the differentiation of killer whale ecotypes in sympatry.

## Methodology

**Figure 1:**
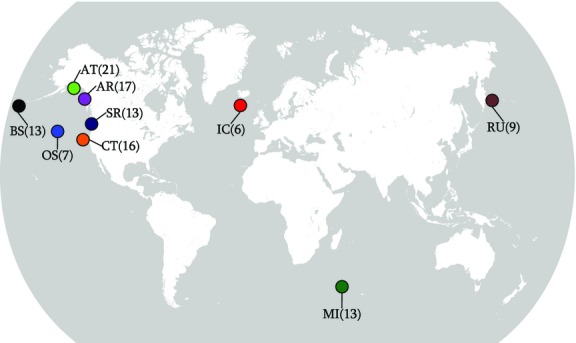
Map of sample sites (colour coded online to match Fig4) and sample sizes parenthetically. Location abbreviations are as defined in Table2. See text for definitions of population codes.

Samples were used from a long-term DNA archive built from previous studies (Hoelzel *et al.* 2007). Newly obtained samples from a population in the Southern Ocean at MI were collected through remote biopsy sampling, using protocols approved by the University of Pretoria's Animal Use and Care Committee (EC023-10) and under permit from the Prince Edward Islands Management Committee. Details on sample numbers and origins are provided in Table S1 (Supporting information). The distribution of sample sites is illustrated in Fig.1.

### RAD sequencing

A modified RAD Seq protocol (Baird *et al.* 2008) was carried out using the *Not*I restriction enzyme to obtain genomewide nuclear data (see Moura *et al.* 2014b for details). A subset of the samples (*N* = 43) was used in an earlier study on phylogenomics (Moura *et al.* 2014b). Briefly, genomic DNA (500 ng–1 μg) was digested at 37 °C overnight, followed by 5 min at 65 °C for termination, with fragments isolated with AMPure XP beads (Agencourt). The forward adapter (p5 adapter) was modified to employ 4 adapters for initial ligation, thus facilitating sequencing cluster differentiation (see Moura *et al.* 2014b).

Adapter-ligated fragments were sheared by sonication targeting a 500 bp average fragment size, recovered with AMPure XP beads and end-repaired using a commercial kit (NEB E6050L) following manufacturer's instructions. Fragments were then selected using streptavidin magnetic beads (Dynabeads® M-280 Streptavidin cat no11205D Life Technologies), using 15 min of binding time at room temperature with agitation, and washed 3 times with Tris/Ethylenediaminetetraacetic acid (TE). DNA that bound to the beads was ligated to a universal p7 sequence adapter.

Amplification primers were designed with 8-bp barcodes to allow multiplexing in a single sequencing lane. A single barcoded primer and a universal primer were used to amplify each sample. Cycling conditions included an initial denaturation step at 98 °C for 30 s, followed by 12–14 cycles of one denaturation step at 98 °C for 10 s, annealing at 60 °C for 30 s, extension at 72 °C for 30 s, followed by a final 5-min extension step. Samples were purified with AMPure XP beads which were washed with 80% ethanol and resuspended in 10 mm Tris pH 7.5. A Qubit (Life Technologies) was used for quantity assessment, and a Bioanalyser 2100 (Agilent) for quality assessment. A 1.5% Pippin prep cassette (Sage Scientific) was used to select pooled samples based on size and quantified by qPCR (KAPA). Two initial 2 × 150 MiSeq runs were carried out without phiX, for a pool of 5 libraries (one sample per library) using this modified and the original Baird *et al*. (2008) published approach, for quality control (see supplement to Moura *et al.* 2014b for more detail). The two methods provide comparable results. Sequencing of the pooled libraries was performed on the Illumina HiSeq 2000 using v3 chemistry.

### SNP mapping and genotyping

Trimmed short reads were mapped against the killer whale genome version 1.1 (GenBank accession no. ANOL00000000) using BWA short read mapper (Li & Durbin 2009). SNP genotypes were called with the Genome Analysis Toolkit (gatk) software package (McKenna *et al.* 2010), using the Unified Genotyper module (DePristo *et al.* 2011). A multisample genotype calling was carried out, including only variable SNPs that were called with a minimum base quality score of 10. vcftools (Danecek *et al.* 2011) was then used to remove all indels, as well as positions with average coverage below 20× and genotype quality below Q20. SNPs for which at least one individual had missing data were removed from the datafile (so that we included only the SNPs that had been confidently scored for all individuals; input file provided on Dryad).

#### Selection detection and population structure analyses

The *F*_ST_ outlier method implemented in lositan (Antao *et al.* 2008) was used to detect putative signs of selection. The following ecotypes/geographic locations were used to define populations (following the results obtained in Hoelzel *et al.* 2007; Parsons *et al.* 2013): Marion Island (MI), Iceland (IC), North Pacific Offshores (OS), Alaskan residents (AR), Southern residents (SR), Alaska transients (AT), California transients (CT), Bering Sea residents (BS) and Russian residents (RU). See Table S1 (Supporting information) for ecotype designation for each putative population. Baseline mean neutral *F*_ST_ was calculated excluding putative selected loci, which were detected using the bisection algorithm over 50 000 simulations with a false discovery rate of 0.1. Fixed differences were identified from genotype profiles and tested against chance using a binomial distribution test.

Comparisons of population structure based on SNPs were carried out using the discriminant analysis of principal components (DAPC) method (Jombart *et al.* 2010) implemented in the software package adegenet (Jombart & Ahmed 2011) both by assigning each individual to the predefined populations, and by determining the best supported number of clusters (1–20) without a priori identification of structure. In both analyses, the first 40 principal components were retained, as were the first four discriminant functions. Patterns of population structure obtained with both neutral and positively selected SNPs were analysed using factorial correspondence analysis (FCA) as implemented in adegenet. Patterns of population diversity and differentiation between neutral and selected SNPs were investigated by comparing pairwise *F*_ST_ and nucleotide differences, using the software arlequin (Excoffier *et al.* 2005). Differential patterns were assessed using the nonparametric Mann–Whitney *U*-test and χ^2^ in 2 × 2 contingency tables (further details provided in the Results section).

#### Functional annotation of relevant SNPs

To identify sites within the relatively well-annotated bottlenose dolphin genome, a sufficient region of sequence to permit confident alignment (2000 bp) centred by the SNP of interest was retrieved from the killer whale genome and mapped against the bottlenose dolphin genome (version 1.69) using the ensembl blat alignment tool. Functional annotation of the genes found to be physically linked to any of these SNPs was carried out using the david web tool (Sherman *et al.* 2007). This analysis was carried out for all SNPs found to have fixed differences between populations, and for the SNPs with the strongest evidence for either positive or balancing selection based on the analysis using lositan (limited to those assigned as an outlier with a probability of 1). Genes of interest were identified as those within 5 kb of a SNP that was either a strong outlier or one of the fixed differences. A minimum linkage block size of 5 kb has been suggested in some studies (e.g. Nash *et al.* 2005; Shimada *et al.* 2011). Due to potential inbreeding and small effective population sizes, linkage blocks in killer whales may be relatively large, but the actual size is unknown. Therefore, for our study, 5 kb was chosen as a compromise: short enough for the SNP to be very likely within a linkage block, and long enough to include a reasonable number of loci to investigate.

To test whether any functions were overrepresented for the genes linked to SNPs putatively under selection, we carried out the gene ontology (GO) term enrichment analysis as implemented in the software fatigo+ (Al-Shahrour *et al.* 2007), using the Babelomics portal (Medina *et al.* 2010). This software compares the proportional representation of a given GO term in the submitted gene list to the proportion in a reference genome. Significance was assessed by means of a two-tailed Fisher's exact test, and only those results still significant at the *P* < 0.05 level after correction for type 1 errors were included.

Enrichment analyses were carried out against the human genome background list, with any duplicate entries removed. We chose the human background list because this is the mammalian genome with the most complete and robust level of annotation. However, we also assessed enrichment against a background list of genes linked to the SNPs identified from our RAD analysis. First we identified genes within 5 kb of all SNPs identified in our RAD analysis, and from the list of genes associated with SNPs identified as neutral (identified using lositan; see above), generated 10 random lists of the same length as the putative selected loci list. These were investigated for the overrepresentation of GO terms using fatigo+ using the human background list. On the assumption that the full RAD SNP-associated list would provide a representation of the background GO terms found across the full genome (though at a lower resolution), we also compared our putative selected loci list against GO terms for genes associated with the full set of RAD SNP loci.

#### Demographic analyses

To investigate killer whale demographic history, we analysed neutral SNPs using diffusion approximation of the joint allele frequency spectrum in ∂a∂i version 1.6.3 (Gutenkunst *et al.* 2009). To polarize the SNPs and determine the derived allele, killer whale reads were mapped against the bottlenose dolphin (*T. truncatus*) genome assembly version 1.69 available from the ensembl database. SNPs were called and filtered as described above, and sites that were found to be under positive or balancing selection were removed. A statistical correction for errors in the outgroup *Tursiops* sequence was applied using a trinucleotide substitution matrix from Hwang & Green (2004) and an estimate of 1.82% sequence divergence between the dolphin and killer whale. Our estimate of sequence divergence was based on a genome size of 2.3 Gb, a divergence time of 10 million years (following the TMRCA between the two species as determined from a multilocus phylogeny, McGowen *et al.* 2009), and a mutation rate of 2.34 × 10^−8^ substitutions/nucleotide/generation, following the mutation rate estimates for the nuclear genome in Odontocetes (Dornburg *et al.* 2012).

The ∂a∂i software is effectively limited to analysing up to three populations at a time. Therefore, we focused on two trios of populations that reflected the most informative lineage histories as indicated by the topology in our phylogenies from Moura *et al*. (2014b). The first analysis included the Southern Ocean (MI), Alaska transient (AT) and OS populations, and the second included AT, OS and the Alaska resident population (AR). We selected AT and AR for these analyses because they represented the largest samples for the transient and resident ecotypes, respectively, and are found in sympatry. In our model, the ancestral population was allowed to vary in population size until the initial population split, and then the two daughter populations were allowed to vary in size with symmetric migration, until the second divergence, after which all three populations were allowed to vary in size and experience symmetric migration. To investigate the order in which populations split, we shuffled the order of the populations for each analysis and then selected the model that demonstrated the maximum likelihood. We then performed 25 independent replicates for that model, chose the maximum-likelihood outcome and report the estimates for all of the parameters under that model (shown as ‘estimate’ in Tables S2 and S3, Supporting information). We assessed uncertainty by conducting 100 bootstrap replicates with replacement for that model, optimizing parameters for 25 iterations each. Bootstrap parameter distributions were mostly right skewed, and therefore, we report median values together with parametric parameters for log(10)-transformed data (mean and 95% confidence intervals). We discarded runs that failed to converge (after Excoffier *et al.* 2013), including all runs that failed to complete or where parameter values were continually increasing. Parameter estimates were converted to more meaningful demographic units by applying the mutation rate specified above, a sequence length of 1.45 Mb and a generation time of 25.7 years (Taylor *et al.* 2007).

## Results

### Neutral population structure

**Figure 2:**
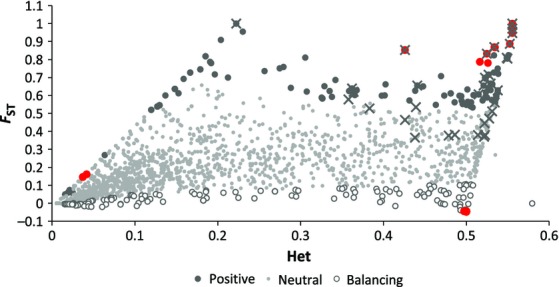
lositan plot identifying outlier SNPs based on neutral expectations. X's represent loci with fixed differences between two or more populations. Black dots (red online) indicate the best supported outlier loci (used for the gene ontology analyses). Het stands for heterozygosity.

**Figure 3:**
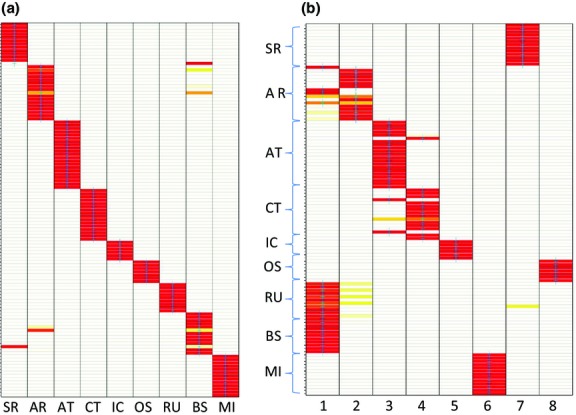
Discriminant analysis of principal components for neutral loci with (a) population identity assigned and (b) when the programme assigns clusters (see text for detailed parameters). Each bar represents a different individual, and darker grey (red online) indicates stronger assignment. Location abbreviations are as defined in Table2.

**Figure 4:**
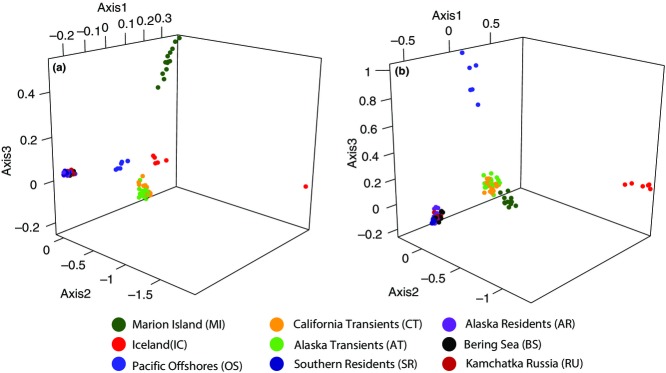
Factorial correspondence analysis of (a) neutral loci and (b) positive outliers. See online for a colour-coded version of the figure.

**Figure 5:**
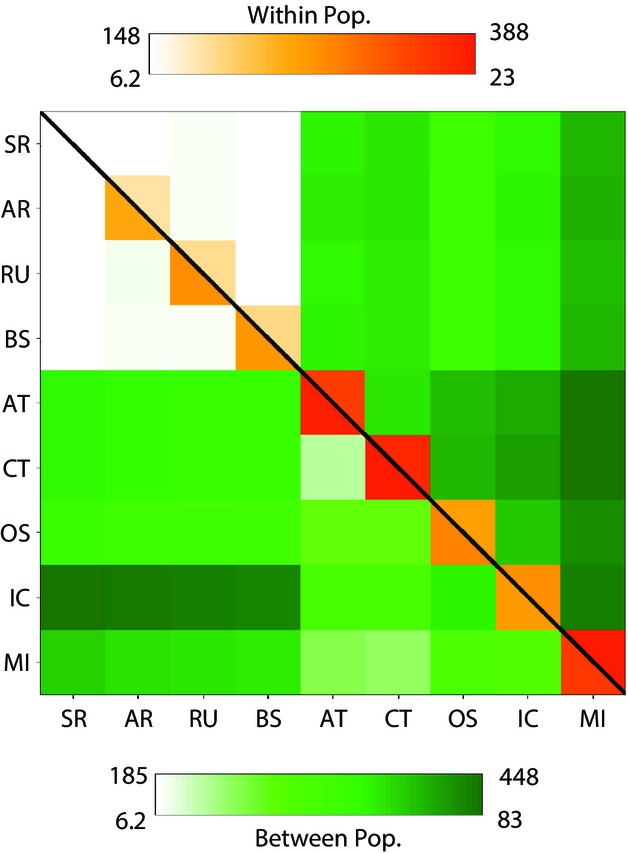
Heat map comparison of the number of allelic differences between neutral (above diagonal) and positively selected SNPs (below diagonal) within (along diagonal) and between populations identified in Fig.1. The grey-scale bars (colour online) indicate the range of values for neutral (above the scale bars) and outlier (below) SNPs.

A total of 3281 variable SNPs could be confidently mapped to the killer whale reference genome (see Methodology), shared among 115 individuals (Genbank accessions: SAMN03020306–SAMN03020378; SAMN02820869–SAMN02820892; SAMN02820894–SAMN02820911). Using lositan (Antao *et al.* 2008), 347 SNPs were identified as outliers, 168 as being potentially under positive selection and 179 as being under balancing selection (Fig.2). Discriminate analysis of principal components (DAPC, Jombart *et al.* 2010) using only the neutral SNPs were able to correctly discriminate all ecotypes and geographical populations when population identity was included as a factor (Fig.3a). When there was no a priori population identification, only the Russian and Bering Sea resident populations grouped together (Fig.3b). From both *F*_ST_ (Table1) and FCA (Fig.4a), a hierarchical structure becomes apparent, such that ecotype clusters were more strongly identified than the differences among populations within ecotypes (Table1, Fig.3a). However, FCA could still discriminate population structure within ecotypes when they were analysed on their own (Fig. S1, Supporting information). This pattern is reinforced by comparisons based on pairwise allelic differences (Fig.5). Figure5 provides a heat map representation of this metric for all pairwise population comparisons and shows the distinction between comparisons among populations based on neutral loci (above diagonal) and those potentially under selection (below diagonal).

**Table 1:** *F*_ST_ comparisons among populations where *N* > 10 for positive outlier loci (upper diagonal) and neutral loci (lower diagonal)

	SR	AR	BS	AT	CT	MI
SR	0	0.2475	0.21784	0.72847	0.73832	0.79715
AR	0.1632	0	0.05128	0.67336	0.6764	0.73618
BS	0.1363	0.04735	0	0.64762	0.64937	0.71197
AT	0.29719	0.26584	0.23356	0	0.05099	0.31034
CT	0.29339	0.25936	0.22429	0.0346	0	0.25045
MI	0.33442	0.30703	0.27442	0.17709	0.15502	0

All comparisons are highly significant (*P* < 0.001). Abbreviations are as defined in Table2.

#### Demographic inference

**Figure 6:**
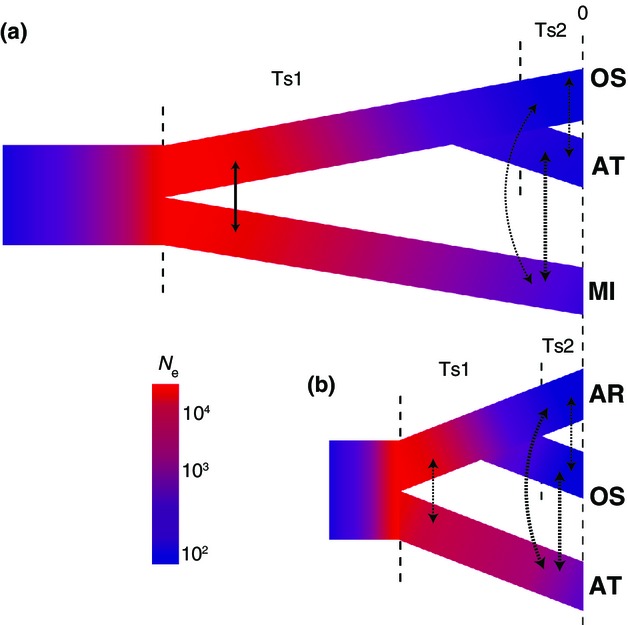
Analysis of demographic history of killer whales. (a) Analysis including Southern Ocean Marion Island (MI) population, Alaska transients (AT) and Offshores (OS). (b) Analysis including AT, OS and Alaska residents (AR). Grey-scale (colour online) approximately represents population size according to the key. Solid lines indicate migration rate >1 individual per generation, and stippled lines indicate rates <1, with thickness approximately representing migration rate. See Tables S4 and S5 (Supporting information) for parameter estimates. Ts stands for time segment, and Ne stands for effective population size.

We assessed demographic history using neutral loci to better understand the process of differentiation among these populations. Our model testing in ∂a∂i (Gutenkunst *et al.* 2009) compared two trios of populations, MI, Alaskan transients (AT) and OS; and AT, OS and AR (see Methodology). MI was the ancestral population associated with the maximum likelihood for the first trio compared, and AT for the second (Tables S4 and S5, Supporting information; consistent with Moura *et al.* 2014a,b). Demographic analyses suggest that the common ancestor of MI, AT and OS was initially small and then grew before the MI population split. Both lineages then continued to grow until later declining, with the AT/OS lineages also declining (Fig.6a; see Table S2, Supporting information for Ne values and confidence limits). Migration during the two population period was relatively high and then declined after the split into three populations (Table S2, Supporting information).

In the second trio (Fig.6b; Table S3, Supporting information), the ancestral population grew until the split between AT and OS/AR, then AT declined (similar to the result from the first trio comparison). The OS/AR lineage grew until the split when both OS and AR declined. Migration was again higher during the 2-population phase, declining after that. The time frame of all modelled population divisions is estimated to be within the late Pleistocene or the Holocene (Tables S2 and S3, Supporting information), with the most recent period reflecting population decline and reduced migration. Details of all parameter estimates are given in Tables S2 and S3 (Supporting information), allele frequency spectra for maximum-likelihood (ML) runs are shown in Figs S2 and S3 (Supporting information), and the model fit is shown in illustrations in Figs S2 and S3 (Supporting information), with the residuals (rows 3 and 4) indicating a good fit.

#### Patterns of differentiation comparing neutral and outlier SNPs

SNPs identified as being under positive selection (showing high *F*_ST_ for a given level of diversity) generated distinct patterns of population structure compared to neutral markers. The FCAs of neutral loci suggest shared kinship between OS, the Icelandic population and transients, as suggested in earlier studies (Pilot *et al.* 2010), with the MI population being most differentiated. For the positive outlier loci, OS and Iceland become more differentiated and MI and transients more similar (Fig.4). We tested for quantitative evidence of distinct differentiation patterns comparing neutral and outlier loci, with respect to the two main ecotypes in the North Pacific, residents and transients. For measures of *F*_ST,_ we consider the following ratio for all possible pairwise combinations: the value of *F*_ST_ for population comparisons between resident and transient ecotypes/the value of *F*_ST_ for population comparisons within resident or transient ecotypes. For neutral loci, this average ratio is 4.15 (range = 1.34–8.59, *N* = 24), significantly lower than when only outlier loci are included (8.18, range = 2.61–14.48, *N* = 24, *Z* = −2.96, *P* = 0.003; Mann–Whitney *U*-test).

This same pattern was evident based on pairwise nucleotide differences (as illustrated in Fig.5) such that the magnitude of the ratio between ecotypes compared to within ecotypes was greater for the outlier loci (3.6-fold compared to 1.6-fold; χ^2^ = 51.06, *P* < 0.0001, d.f. = 1; Yate's correction applied). Comparing residents and transients against the other three groups combined (OS, IC and MI) for pairwise nucleotide differences also showed a distinct pattern for neutral compared to outlier loci. There was greater similarity between residents and the outgroups for neutral markers, but greater similarity between transients and the outgroups for outlier loci (Fig.5; χ^2^ = 75.4, *P* < 0.0001, d.f.  = 1).

Fixed differences (marked with an ‘X’ in Fig.2) were found for both neutral and positive outlier loci in pairwise comparisons between putative populations, but were much more common for outlier loci (Table2). This will likely include outliers due to both selection and drift (see SNP mapping and genotyping). Overall, there were 33 SNPs showing fixed differences for one or more population comparisons; however, most sample sizes were small, and so only some showed this more often than may occur by chance (Tables2 and S6, Supporting information). From FCA of these SNPs, a pattern of four population clusters emerged: the North Pacific resident populations, the North Pacific transients together with the MI whales, the OS and the Icelandic sample (Fig. S4, Supporting information). There were more fixed difference loci than expected by chance (Table2) for comparisons between populations from each of these clusters; however, the number between residents and transients was relatively low and poorly supported. Among resident populations, SR (the ‘southern residents’ from Washington State waters) was most differentiated (Table2) and also showed the lowest level of within-population differentiation for both neutral and outlier loci (Fig.5).

**Table 2:** Number of fixed differences between populations

	SR	AR	BS	RU	OS	AT	CT	IC	MI
SR (13)		0	0	0	8^*^	7^*^	5^*^	15^*^	9^*^
AR (17)	0		0	0	5^*^	3	2	12^*^	6^*^
BS (13)	0	0		0	5^*^	3	2	9^*^	5^*^
RU (9)	0	0	0		4^*^	3	2	7^*^	4^*^
OS (7)	5^*^	1	0	0		1	1	7^*^	2
AT (21)	0	0	0	0	0		0	1	0
CT (16)	0	0	0	0	0	0		0	0
IC (6)	6^*^	4^*^	3	3	2	0	0		0
MI (13)	2	0	0	0	0	0	0	0	

Sample size given as (*N*). Neutral loci on lower diagonal, outliers (for positive selection) on upper diagonal. Comparisons that show a significant number of fixed differences beyond that expected from sampling effects by chance are shown with an asterisk. SR, southern (Washington State) residents; AR, Alaskan residents; BS, the Bering Sea residents; RU, the Kamchatka, Russia residents; AT, Alaskan transients; CT, Californian transients; IC, Iceland; MI, Marion Island; OS, Offshore.

#### Functional annotation of outlier and fixed difference SNPs

We considered possible linkage to known genes for the most extreme outliers (red dots in Fig.2; assigned as an outlier with a probability of 1; see Methodology) for loci under putative positive or balancing selection. Of 21 SNPs identified for balancing selection, only 3 loci were closely linked (defined here as within 5 kb, see Methodology) to a known gene, and these were associated especially with cell signalling (RAB43, BHLHB9, GRM7). Among those identified for positive selection, a higher proportion (11/19) were linked to genes associated with functions relevant to reproduction, growth and metabolism, among others (see Table S7, Supporting information). The genes linked to fixed difference SNPs again represented loci relevant to aspects of physiology, growth and reproduction (Table S8, Supporting information). There was just one locus where the fixed difference represented a nonsynonymous change within an exon. This was for GATA4 (at residue 187 changing a proline to a glutamine), a transcription factor involved in various functions including the development of cardiac tissue (Hu *et al.* 2010) and testes size/reproductive function (Kyrönlahti *et al*. 2011). The fixed difference at this locus was between the resident populations and all others.

We then considered the GO of these loci (all 24 unique loci identified as under strong positive selection or reflecting fixed differences as identified above) in the context of the proportional representation of GO terms compared to what may be expected by chance from a fully annotated reference genome (using the human genome due to the high-quality annotation; Table S9, Supporting information). The results show that 30 terms are significantly enriched compared to what would be expected by chance, including terms associated with the digestive and circulatory systems. Significantly enriched functions and the associated genes are listed in Table S9 (Supporting information), and the relevant genes marked in bold text in Tables S7 and S8 (Supporting information). Two transcription factor genes were associated with multiple enriched functional terms, GATA4 and MZF1.

Our tests for overrepresentation of GO terms in 10 random lists of genes linked to neutral loci (each of the same length as the list of genes putatively under selection) found no evidence of overrepresentation for a *P*-value after correction for false discovery of either 0.05 or 0.10. When we used genes associated with all SNP loci identified from our RAD analysis as the background instead of the full human genome, only four terms were overrepresented at a *P*-value of 0.05, but 32 at a *P*-value of 0.10, indicating that this comparison provided reduced power. GATA4 and MZF1 remained associated with multiple enriched terms.

## Discussion

### Population structure and demographic analyses

Summary statistics (*F*_ST_ and pairwise differences) and ordination analyses (DAPC and FCA) show strong differentiation both among ecotypes and among geographic populations within ecotypes at neutral loci, providing a finer level of resolution to the patterns found earlier in microsatellite DNA studies (Hoelzel *et al.* 2007; Parsons *et al.* 2013). In particular, in contrast to the earlier microsatellite DNA studies, all putative populations, including four parapatric resident populations within the North Pacific, can be genetically distinguished.

There was also evidence for hierarchical clusters that had not been clearly defined using microsatellite DNA data and that sometimes differ from inference previously gained from mtDNA genetic lineages (Hoelzel *et al.* 2002; Morin *et al.* 2010; Moura *et al.* 2014b). This inconsistency between inference from mtDNA and nuclear markers (e.g. a level of mtDNA similarity between residents and OS not seen for nuclear markers) had been noted in earlier studies using alternative methods (Pilot *et al.* 2010; Moura *et al.* 2014b), and is likely due to stochastic factors affecting the mtDNA lineages. The most coherent ordination clusters grouped populations by resident or transient ecotype in the North Pacific. Offshores, Iceland and MI were all clearly differentiated from the resident and transient clusters based on neutral loci. Demographic analyses concurred with the ordination analyses, as well as previous studies, indicating low migration rates between the sympatric transient and resident ecotypes.

Our temporal estimates of population differentiation (Tables S2 and S3, Supporting information) are broadly consistent with earlier microsatellite DNA estimates, suggesting that populations in the North Pacific differentiated during the relatively recent past (during the late Pleistocene and Holocene, Hoelzel *et al.* 2007). They support previous studies suggesting a higher mutation rate (3%/MY for the mitogenome; Ho & Lanfear 2010; Moura *et al.* 2013b) than used in published estimates of the time of initial division between transients in the North Pacific and other lineages worldwide (702 ka based on 0.3%/MY; Morin *et al.* 2010; Foote *et al.* 2011). Our calculation of divergence times is based on a published estimate for the average substitution rate in the Odontocete nuclear genome (see Methodology) and is more consistent with inference using the faster mtDNA mutation rate estimate. The timing is consistent with a signal for a population bottleneck during the last glacial period, as indicated from whole-genome analyses for individual killer whales from the North Pacific and North Atlantic (Moura *et al.* 2014a).

The demographic data presented in Fig.6 suggest a smaller ancestral population (possibly postbottleneck; Moura *et al.* 2014a) that was expanding at the time of the division between the Southern Ocean and North Pacific transient populations. If new habitat was being released (as would be the case following a glacial period) at the time of the establishment of the nearshore (resident) piscivorous ecotype, then this would be consistent with the evident later reduction in effective population size as founder populations were established. Low gene flow, kin associations in social groups, and the expansion of these new populations along matrilines would reinforce and exaggerate the degree of differentiation between the source and the newly established populations. The relatively high estimated level of gene flow after the initial split between MI and AT, and the subsequent reduction in gene flow, are consistent with this scenario and also consistent with recent divergence within the North Pacific. Inference from allele frequency spectra (as implemented in ∂a∂i) has known limitations (Myers *et al.* 2008), but also empirical support in comparison with known geologic or historical events (Gutenkunst *et al.* 2009; Zhao *et al.* 2013). In our case, comparable values are similar to those obtained by different methods in earlier studies (e.g. Hoelzel *et al.* 2007; Moura *et al.* 2014a).

### Differentiation at putative selected SNPs

The pattern of differentiation among putative populations changes when positive outlier loci are considered on their own. For example, there is increased similarity among separate populations within each of the resident and transient ecotypes, and significantly greater differentiation between these two ecotypes (see Table1 and Fig.5). A similar pattern was seen for genotypes associated with freshwater or marine habitat in sticklebacks, such that loci under selection were more similar within ecotypes (Hohenlohe *et al.* 2010). However, the method chosen to identify outlier loci (lositan) has a high false discovery rate (Antao *et al.* 2008), which means that there are likely neutral loci among those identified as being under selection (while the identification of neutral loci should be relatively free of loci under selection). Therefore, the observed pattern could be expected either when loci under selection reflect genes that are associated with adaptation by ecotype, or if strong drift generates differentiation between ecotypes more than among other populations, or by some combination of both factors.

Because ecotype populations are among those used in identifying the outliers, an exaggerated pattern of drift could be seen in the outliers, although this effect should be similar for neutral loci. However, the comparison of each North Pacific ecotype (residents and transients) against outgroups (MI, Iceland and the OS) provided additional inference that there was exaggerated differentiation for outliers which more likely reflects differential selection among ecotypes. In this case, the outliers showed the opposite relationship to the outgroups compared to the neutral markers (Fig.5). It is also the case that strong outliers and fixed differences were associated with genes that have ecologically relevant functions (Tables S7–S9, Supporting information). Because of the potential for neutral loci being grouped with the outliers, we considered GO terms only for those outliers that were strongly supported (as identified by the program lositan, see Methodology), or associated with fixed differences between populations.

Killer whale ecotypes forage on different resources (although some populations may prey on multiple resource types; see Best *et al.* 2010; Foote *et al.* 2013), may have different metabolic requirements as a consequence and have different social structure (e.g. Ford *et al.* 1998; Beck *et al.* 2012), which may have implications for reproductive strategy including the potential for sperm competition. A total of 24 loci potentially under positive selection linked to SNPs were identified, and some of these did have functions consistent with relevant aspects of digestive physiology and reproductive biology (see Tables S7 and S8, Supporting information). Because this species has a demographic history involving population bottlenecks (see Hoelzel *et al.* 2002; Moura *et al.* 2014a), and a social structure that promotes kin association (although mating is typically outside of the social group, and kinship within social groups not substantially different than among regional social groups; Pilot *et al.* 2010; Ford *et al.* 2011), linkage blocks may be long. However, as an assessment of enrichment showed that some GO terms associated with genes from our list were significantly overrepresented (Table S9, Supporting information), the implication is that we are identifying loci that are relevant to selection. This is reinforced by the fact that random lists of genes linked to neutral SNPs showed no evidence for enrichment of GO terms. For the list of genes putatively under selection, there was significantly stronger overrepresentation than expected by chance for 30 GO terms, including terms associated with digestion (consistent with one key distinction among ecotypes).

We used the human genome as a reference as it is the best annotated mammalian genome and GO terms should be mostly species neutral (see Primmer *et al.* 2013). At the same time, the choice of an appropriate background reference list is important (Primmer *et al.* 2013), and in this case we contend that the full set of mammalian genes is appropriate, even though the outliers and fixed difference SNPs were chosen from among the loci identified from a subsample of sites provided by the RAD data. This subset provides a list that is expected to be representative, but is an order of magnitude smaller, and therefore only an approximate representation. When we compared against the RAD list, we found that there is lower power as expected, and although some GO terms associated with the same subset of genes are identified as potentially overrepresented, the resolution is too low for strong inference. When using the full human reference, there is a risk that enrichment would represent false positives, but that would imply finding enriched terms for our sets of random neutral loci, which we did not observe.

In general, differential patterns for positive outliers compared to neutral loci imply selection (for at least some loci among the outliers), although it was not possible to fully determine the nature of the adaptive differences. Distinct structure at outlier loci included MI being more similar to the transient populations, and Iceland (and to some extent OS) being more distinct from the residents. These are in each case comparisons within broad ecotype categories (as MI whales take pinnipeds as part of their diet as do transients, while OS, Icelandic and resident whales all feed on fish, see review in de Bruyn *et al.* 2013). While the implication from the increased similarity between transients and MI is consistent with shared ecotype, increased differentiation among the three fish-eating groups implies that there are additional factors beyond prey choice involved in the adaptation of these various populations to their different habitats (although phenotypic variation among these populations is not strong, and we do not know enough about the relevant genes to speculate further). The possibility of changing specializations over time in the North Atlantic would potentially be relevant (Foote *et al.* 2013). More generally, it is clear that it will be important to learn more about the characteristics that may reflect ecotype adaptation. Increasingly, studies have found that key differences in phenotype are likely to be related to gene regulation (Carroll 2005), and some of our results (e.g. with respect to GATA4 and MZF1) could be consistent with that.

### Fixed differences and the question of taxonomy

Some further insight can be gained through the analysis of fixed differences among populations. These are differences that could be sustained either through an extended period of isolation and drift, by inbreeding, by strong selection or by sampling effects from small sample sizes. From our data, there is much stronger evidence for fixed differences at outlier than neutral loci, but these will likely include loci that are outliers due to drift as well as selection. The pattern of differentiation at the 33 fixed loci (based on FCA) emphasizes the clusters defined by the outlier loci in general. Transients cluster with MI, residents group together, and both OS and Iceland form independent clusters (Fig. S4, Supporting information, Table2). Among the fixed differences, 20 were within 5 kb of a functional locus with 16 being within or just outside the gene. The GO terms indicate functions associated with digestion, nutrient metabolism, protein metabolism, body development and reproduction (among other potentially relevant functions; see Table S8, Supporting information).

Just one locus showed a fixed difference at a nonsynonymous change within an exon, the transcription factor GATA4, and this reflected a fixed difference between the residents and all other groups. Among its various functions, GATA4 is involved in testis development in mice (Kyrönlahti *et al*. 2011). The highly stable social groups and apparent strategy for mating in temporary assemblages (Pilot *et al.* 2010) within the resident populations may be consistent with sperm competition, possibly promoted by changes at this locus. However, there are few relevant data on sperm competition in this species, and among 31 cetacean species the killer whale was ranked 13th for residual testes size (used as an index for the potential for sperm competition; MacLeod 2010). More work is needed to further explore the potential role of sperm competition.

The presence of fixed differences can raise questions about alpha taxonomy, although there is at present no clear expectation for what the proportion of fixed differences that defines a species-level difference might be, and the range of observed values is broad. Empirical observations for allozyme markers suggested that distinct species can be expected to exhibit fixed differences at approximately 15% of loci analysed (O'Donnell *et al.* 2004). However, a genomic study comparing two closely related flycatcher species of the genus *Ficedula* (with a 2 Ma most common recent ancestor, but still exhibiting limited hybridization) found that only 1.8% of all segregating sites identified were fixed between the two species (Ellegren *et al.* 2012). The percentages of fixed differences between killer whale populations were typically much smaller (e.g. average values for resident populations vs. Iceland = 0.328%; residents vs. all others = 0.177%; residents vs. transients = 0.103%). There is only one SNP showing a fixed difference between all individuals of one ecotype (residents) and all others, and that was GATA4 (which also shows a nonsynonymous change). The SR show more pronounced fixed differences (Table2) and less differentiation within the population compared to other putative populations (Pilot *et al.* 2010; Fig.5). Earlier studies using nonequilibrium models showed support for gene flow between residents and transients following a point of division (Hoelzel *et al.* 2007); however, our RAD data suggest that this may have been mostly during the period soon after division (consistent with the temporal data about migration shown in Table S3, Supporting information).

## Conclusions

As discussed in earlier studies (Hoelzel *et al.* 2007), regional killer whale populations can be small and evidently composed of an extended matriline, likely founded by one or a few stable social groups. The SR are an extreme example of this. For this reason, it is appropriate to be cautious about the interpretation of fixed differences, as these could be promoted relatively quickly by drift when Ne is small. Earlier studies have proposed everything from relatively recent (Holocene) population differentiation (e.g. Hoelzel *et al.* 2007) to species-level differences established in the middle Pleistocene (Morin *et al.* 2010), and we do not propose to fully resolve the question of alpha taxonomy here. However, the time frame implied from our RAD data and from the demographic profiles generated from nuclear genomes (Moura *et al.* 2014a) suggests relatively recent events. The data on population structure based on outlier loci and the significant overrepresentation of biologically relevant GO terms suggest that natural selection has contributed to the degree of differentiation among populations of different ecotypes in the North Pacific. Taken together, these data suggest that differentiation in sympatry is based in part on ecological processes, but that differentiation is likely being facilitated by the life history of killer whales, founder events and differentiation by drift. Both the demographic analyses and the small proportion of fixed differences suggest that if incipient speciation is the correct interpretation, it began very recently and is ongoing.

## Notes

A.R.H. designed the study, A.E.M. and A.R.H. carried out the analyses, interpreted the results and wrote the manuscript, J.G.K., R.C., M.A.H. and N.H. produce the genomic data used in the study, A.W. contributed to the analyses and writing, R.R.R., P.J.N.B. and M.E.D. contributed with biological material and killer whale expertise, and all authors read draft versions of the manuscript.

## Data accessibility

DNA sequences: Genbank accessions: SAMN03020306–SAMN03020378; SAMN02820869–SAMN02820892; SAMN02820894–SAMN02820911. VCF files for SNP data: Dryad doi: 10.5061/dryad.qk22t.

## Supporting Information

Table S1 Number of samples used in this study by predefined population. **Table S2** Demographic estimates from ∂a∂i for the MI/AT/OS analysis. **Table S3** Demographic estimates from ∂a∂i for the AT/OS/AR analysis. **Table S4** Model comparisons for order of population splitting for the MI, AT, OS analysis. **Table S5** Model comparisons for order of population splitting for the AT, OS, AR analysis. **Table S6** Probability that observed fixed differences between population pairs were significant (bold and italics). **Table S7** Genes listed by putative functions based on GO terms linked to strong outlier SNPs for positive selection (genes in bold show significant over-representation compared to reference GO terms; see Table S9). **Table S8** Genes listed by putative functions based on GO terms linked to SNPs associated with fixed differences among putative populations (genes in bold show significant over-representation compared to reference GO terms; see Table S9). **Table S9** List of significantly enriched GO terms as compared to the proportions in the human reference genome. **Fig. S1** (a) FCA of resident populations on their own using neutral markers and (b) the same for transient populations. **Fig. S2** Pairwise comparisons of the allele frequency spectrum of the observed data (top row) versus the model (second row) and plots of the residuals (third and fourth rows) from ∂a∂i for the MI, AT, OS analysis. **Fig. S3** Pairwise comparisons of the allele frequency spectrum of the observed data (top row) versus the model (second row) and plots of the residuals (third and fourth rows) from ∂a∂i for the AT, OS, AR analysis. **Fig. S4** Individual FCA plot using the 33 SNPs that were fixed for a single allele in at least one a priori population.
