# Supplementary material for: Population genomics of the killer whale indicates ecotype evolution in sympatry involving both selection and drift
Source: Mol Ecol. 2014 Oct 12;23(21):5179–92. doi: 10.1111/mec.12929 (PMC4237148; doi:10.1111/mec.12929)

**Supplementary Materials**

Table S1: Number of samples used in this study by predefined population

| **Population Code** | **Ecotype** | **Sampling Location** | **Number of samples** |
| --- | --- | --- | --- |
| AR | Resident | Alaska | 17 |
| SR | Resident | Washington State | 13 |
| RU | Resident | Kamchatka, Russia | 9 |
| BS | Resident | Bering Sea | 13 |
| AT | Transient | Alaska | 21 |
| CT | Transient | California | 16 |
| OS | Offshore | Eastern North Pacific | 7 |
| IC | NA | Iceland | 6 |
| MI | Antarctic Type B | Marion Island | 13 |

Table S2: Demographic estimates from ∂a∂I for the MI/AT/OS analysis. Column marked ‘estimate’ provides the point estimates from the run with the maximum likelihood, as shown in Table S4. Bootstrap replicate distributions were not normally distributed and so the median of the distribution is presented, along with the mean and 95% upper and lower confidence intervals for the log transformed distribution.

|  |  |  | **Untransformed Bootstraps** |  | **Log Transformed Bootstraps** | | |  |
| --- | --- | --- | --- | --- | --- | --- | --- | --- |
| **Parameter** | **Symbol** | **Estimate** | **Median** |  | **Mean** | **95% Lower** | **95% Upper** | **Unit** |
| Ne ancestor after growth | nuAS | 1947 | 975 |  | 900 | 579 | 1398 | Individuals |
| N_eMI_ after Ts1 | nu1M | 15047 | 5518 |  | 3385 | 2150 | 5329 | Individuals |
| N_eMI_ after Ts2 | nu1F | 426 | 603 |  | 585 | 451 | 758 | Individuals |
| N_eAT/OS_ after Ts1 | nu2M | 4190 | 3653 |  | 2743 | 2006 | 3752 | Individuals |
| N_eAT_ after Ts2 | nu2F | 339 | 374 |  | 391 | 305 | 500 | Individuals |
| N_eOS_ after Ts2 | nu3F | 154 | 170 |  | 178 | 137 | 230 | Individuals |
| Migration between MI and AT/OS after Ts1 | mMid | 2.278 | 1.435 |  | 1.045 | 0.565 | 1.940 | Individuals/Generation |
| Migration between MI and AT after Ts2 | m12F | 0.308 | 0.291 |  | 0.302 | 0.211 | 0.431 | Individuals/Generation |
| Migration between MI and OS after Ts2 | m13F | 0.081 | 0.085 |  | 0.097 | 0.068 | 0.139 | Individuals/Generation |
| Migration between AT and OS after Ts2 | m32F | 0.102 | 0.089 |  | 0.090 | 0.065 | 0.123 | Individuals/Generation |
| Time between initial split (Ts1) and final split (Ts2) | Ts1 | 34337 | 40038 |  | 32352 | 22648 | 46215 | Years before present |
| Time between final split (Ts2) and present | Ts2 | 3849 | 4814 |  | 6543 | 4473 | 9571 | Years before present |

Table S3: Demographic estimates from ∂a∂I for the AT/OS/AR analysis. Column marked ‘estimate’ provides the point estimates from the run with the maximum likelihood, as shown in Table S5. Bootstrap replicate distributions were not normally distributed and so the median of the distribution is presented, along with the mean and 95% upper and lower confidence intervals for the log transformed distribution.

|  |  | **Estimate** | **Untransformed Bootstraps** |  | **Log Transformed Bootstraps** | | |  |
| --- | --- | --- | --- | --- | --- | --- | --- | --- |
| **Parameter** | **Symbol** | **AT/OS/AR** | **Median** |  | **Mean** | **95% Lower** | **95% Upper** | **Unit** |
| Ne ancestor after growth | nuAS | 2522 | 1953 |  | 1798 | 1328 | 2435 | Individuals |
| N_eAT_ after Ts1 | nu1M | 1460 | 1981 |  | 2039 | 1475 | 2819 | Individuals |
| N_eAT_ after Ts2 | nu1F | 937 | 696 |  | 572 | 454 | 722 | Individuals |
| N_eOS/AR_ after Ts1 | nu2M | 4321 | 3512 |  | 4558 | 3341 | 6219 | Individuals |
| N_eOS_ after Ts2 | nu2F | 97 | 184 |  | 175 | 135 | 228 | Individuals |
| N_eAR_ after Ts2 | nu3F | 57 | 108 |  | 102 | 79 | 131 | Individuals |
| Migration between AT and OS/AR after Ts1 | mMid | 0.229 | 0.956 |  | 1.413 | 0.847 | 2.358 | Individuals/Generation |
| Migration between AT and OS after Ts2 | m12F | 0.151 | 0.144 |  | 0.109 | 0.070 | 0.168 | Individuals/Generation |
| Migration between AT and AR after Ts2 | m13F | 0.142 | 0.113 |  | 0.083 | 0.059 | 0.118 | Individuals/Generation |
| Migration between OS and AR after Ts2 | m32F | 0.030 | 0.050 |  | 0.035 | 0.023 | 0.054 | Individuals/Generation |
| Time between initial split (Ts1) and final split (Ts2) | Ts1 | 14689 | 28461 |  | 28723 | 22210 | 37145 | Years before present |
| Time between final split (Ts2) and present | Ts2 | 1832 | 3868 |  | 5124 | 3455 | 7599 | Years before present |

Table S4: Model comparisons for order of population splitting for the MI, AT, OS analysis

|  | **Alternative Models** | | |
| --- | --- | --- | --- |
| Splitting  Pattern | 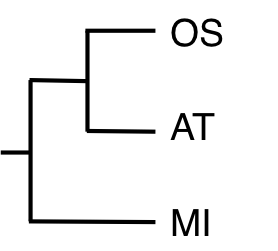 | 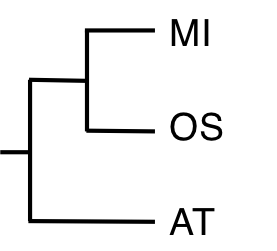 | 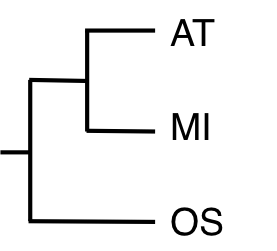 |
| Log likelihood | **-1367.45** | **-1369.52** | **-1375.07** |

Table S5: Model comparisons for order of population splitting for the AT, OS, AR analysis

|  | **Alternative Models** | | |
| --- | --- | --- | --- |
| Splitting  Pattern | 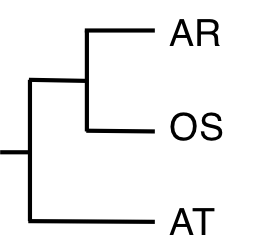 | 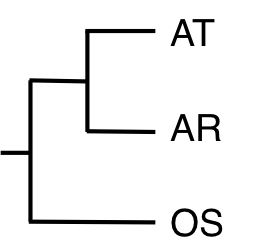 | 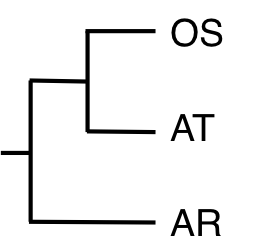 |
| Log likelihood | **-1267.05** | **-1309.71** | **-1278.21** |

Table S6: Probability that observed fixed differences between population pairs were significant (bold & italics). Those that are not significant after Bonferonni correction are shown in grey.

|  | SR | AR | BS | RU | OS | AT | CT | IC | MI |
| --- | --- | --- | --- | --- | --- | --- | --- | --- | --- |
| SR (13) |  | - | - | - | ***0.000000*** | ***0.000000*** | **0.000038** | ***0.000000*** | ***0.000000*** |
| AR (17) | - |  | - | - | ***0.000035*** | 0.0023 | 0.017 | ***0.000000*** | ***0.000005*** |
| BS (13) | - | - |  | - | ***0.000035*** | 0.0023 | 0.017 | ***0.000000*** | ***0.000038*** |
| RU (9) | - | - | - |  | ***0.000257*** | 0.0022 | 0.017 | ***0.000000*** | ***0.000276*** |
| OS (7) | ***0.000034*** | 0.1284 | - | - |  | 0.1288 | 0.1283 | ***0.000000*** | 0.0163 |
| AT (21) | - | - | - | - | - |  | - | 0.1279 | - |
| CT (16) | - | - | - | - | - | - |  | - | - |
| IC (6) | ***0.000004*** | ***0.00026*** | 0.0021 | 0.002 | 0.0156 | - | - |  | - |
| MI (13) | 0.0169 | - | - | - | - | - | - | - |  |

Table S7: Genes listed by putative functions based on GO terms linked to strong outlier SNPs for positive selection (genes in bold show significant over-representation compared to reference GO terms; see Table S9).

| **Number of SNPs** | **General Function** | **Linked Genes** |  |
| --- | --- | --- | --- |
| 2 | DNA binding; DNA processing | **GATA4**, RPRD2 |  |
| 2 | Ion Binding | **GATA4**; **MZF1** |  |
| 2 | Cytoskeleton | ELMOD3; **SEPT5** |  |
| 2 | Digestion, Nutrient metabolism | **GATA4**; **RETSAT** |  |
| 2 | Fertilization, Reproduction | **GATA4**, TEX10 |  |
| 1 | Alternative splicing | NSMCE4A |  |
| 1 | Transcription regulation | **MZF1** |  |
| 1 | RNA Processing | **GATA4** |  |
| 1 | Muscle Development and function | **GATA4** |  |
| 1 | Vesicle transport | ELMOD3 |  |
| 1 | Protein metabolism | **PTPMT1** |  |
| 1 | Heart function and development | **GATA4** |  |
| 1 | Cell cycle and growth | **SEPT5** |  |
| 1 | Motor coordination, Spatial awareness | **SEPT5** |  |
| 1 | Body development | **GATA4** |  |
| 1 | Lipid regulation | XKR6 |  |

Table S8: Genes listed by putative functions based on GO terms linked to SNPs associated with fixed differences among putative populations

(genes in bold show significant over-representation compared to reference GO terms; see Table S9).

| **# SNPs** | **General Function** | **Linked Genes** |  | **Relevant Populations** |
| --- | --- | --- | --- | --- |
| 6 | Alternative splicing | SMCR7; CELF4; NSMCE4A; RPRD2; SF3A3; **UBTF** |  | SR vs OS; SR vs ICE-MI; R vs rest; R-OS vs ICE |
| 5 | DNA binding; DNA processing | **GATA4**; **MZF1**; **PDK1**; **TOP1**; **UBTF** |  | R vs rest; TR-MI-ICE-OS vs SR; OS vs rest; R vs ICE; R vs ICE-MI |
| 5 | Ion Binding | **GATA4**; **TBC1D9**; FHL3; **MZF1**;RNF220; SF3A3 |  | R vs rest; SR-ICE vs AT; R-OS vs ICE; TR-MI-ICE-OS vs SR; AR vs ICE-OS |
| 4 | Cytoskeleton | ELMOD3; FLII; FHL3; **HOOK2** |  | R-OS vs ICE; SR vs OS; R vs OS |
| 3 | Transcription regulation | FLII; **MZF1**; **UBTF** |  | SR vs OS; TR-MI-ICE-OS vs SR; R vs ICE-MI |
| 3 | RNA Processing | **GATA4**; CELF4;SF3A3 |  | R vs rest; SR vs ICE-MI; R-OS vs ICE |
| 3 | Digestion, Nutrient metabolism | **GATA4**; **PDK1**; **RETSAT** |  | R vs rest; OS vs rest; R-OS vs ICE |
| 2 | Fertilization, Reproduction | **GATA4**; CELF4 |  | R vs rest; SR vs ICE-MI |
| 2 | Muscle Development and function | **GATA4**; FLII |  | R vs rest; SR vs OS |
| 2 | Vesicle transport | ELMOD3; **HOOK2** |  | R-OS vs ICE; R vs OS |
| 2 | Immunity | **BST1**, **MAVS** |  | SR vs OS; TR-MI-ICE-OS vs SR |
| 1 | Protein metabolism | **HOOK2** |  | R vs OS |
| 1 | Heart function and development | **GATA4** |  | R vs rest |
| 1 | Cell cycle and growth | **TOP1** |  | R vs ICE |
| 1 | Motor coordination, Spatial awareness | CMTM2 |  | R-OS vs ICE |
| 1 | Body development | **GATA4** |  | R vs rest |

Table S9: List of significantly enriched GO terms as compared to the proportions in the human reference genome.

| **Go Term** | **GO Term Description** | **Function** | **% in Gene List** | **% in Reference** | **Genes** | **Odds ratio** | **p-value** | **Adjusted p-value** |
| --- | --- | --- | --- | --- | --- | --- | --- | --- |
| GO:0000155 | [two-component sensor activity](javascript:void(0);) | Cellular Activity | 8.33 | 0.05 | PDK1,MZF1 | 5.21 | 0.00008 | 0.02124 |
| GO:0004673 | [protein histidine kinase activity](javascript:void(0);) | Cellular Activity | 8.33 | 0.05 | PDK1,MZF1 | 5.21 | 0.00008 | 0.02124 |
| GO:0003917 | [DNA topoisomerase type I activity](javascript:void(0);) | DNA Metabolism | 8.33 | 0.03 | TOP1,MZF1 | 5.81 | 0.00003 | 0.01530 |
| GO:0004439 | [phosphatidylinositol-4,5-bisphosphate 5-phosphatase activity](javascript:void(0);) | Cellular Activity | 8.33 | 0.11 | PTPMT1,MZF1 | 4.43 | 0.00036 | 0.04388 |
| GO:0016799 | [hydrolase activity, hydrolyzing N-glycosyl compounds](javascript:void(0);) | Cellular Activity | 8.33 | 0.07 | BST1,MZF1 | 4.90 | 0.00015 | 0.02955 |
| GO:0007032 | [endosome organization](javascript:void(0);) | Cellular Activity | 8.33 | 0.06 | HOOK2,MZF1 | 4.97 | 0.00013 | 0.03859 |
| GO:0007033 | [vacuole organization](javascript:void(0);) | Cellular Activity | 8.33 | 0.12 | HOOK2,MZF1 | 4.35 | 0.00042 | 0.04598 |
| GO:0007040 | [lysosome organization](javascript:void(0);) | Cellular Activity | 8.33 | 0.1 | HOOK2,MZF1 | 4.47 | 0.00033 | 0.04302 |
| GO:0006360 | [transcription from RNA polymerase I promoter](javascript:void(0);) | DNA Metabolism | 8.33 | 0.05 | UBTF,MZF1 | 5.30 | 0.00007 | 0.03245 |
| GO:0043087 | [regulation of GTPase activity](javascript:void(0);) | Cellular Activity | 12.5 | 0.58 | TBC1D9,RETSAT,MZF1 | 3.20 | 0.00038 | 0.04334 |
| GO:0001523 | [retinoid metabolic process](javascript:void(0);) | Cellular Activity | 8.33 | 0.09 | RETSAT,MZF1 | 4.61 | 0.00026 | 0.03859 |
| GO:0006776 | [vitamin A metabolic process](javascript:void(0);) | Cellular Activity | 8.33 | 0.1 | RETSAT,MZF1 | 4.56 | 0.00028 | 0.03859 |
| GO:0006265 | [DNA topological change](javascript:void(0);) | DNA Metabolism | 8.33 | 0.11 | TOP1,MZF1 | 4.43 | 0.00036 | 0.04334 |
| GO:0001702 | [gastrulation with mouth forming second](javascript:void(0);) | Digestion | 8.33 | 0.08 | GATA4,MZF1 | 4.77 | 0.00019 | 0.03859 |
| GO:0001947 | [heart looping](javascript:void(0);) | Circulation | 8.33 | 0.1 | GATA4,MZF1 | 4.56 | 0.00028 | 0.03859 |
| GO:0048546 | [digestive tract morphogenesis](javascript:void(0);) | Digestion | 8.33 | 0.1 | GATA4,MZF1 | 4.56 | 0.00028 | 0.03859 |
| GO:0048547 | [gut morphogenesis](javascript:void(0);) | Digestion | 8.33 | 0.07 | GATA4,MZF1 | 4.83 | 0.00017 | 0.03859 |
| GO:0048557 | [embryonic digestive tract morphogenesis](javascript:void(0);) | Digestion | 8.33 | 0.03 | GATA4,MZF1 | 5.81 | 0.00003 | 0.02956 |
| GO:0048562 | [embryonic organ morphogenesis](javascript:void(0);) | Development | 8.33 | 0.09 | GATA4,MZF1 | 4.66 | 0.00023 | 0.03859 |
| GO:0055123 | [digestive system development](javascript:void(0);) | Digestion | 8.33 | 0.1 | GATA4,MZF1 | 4.56 | 0.00028 | 0.03859 |
| GO:0006903 | [vesicle targeting](javascript:void(0);) | Cellular Metabolism | 8.33 | 0.09 | SEPT5,MZF1 | 4.66 | 0.00023 | 0.03859 |
| GO:0016079 | [synaptic vesicle exocytosis](javascript:void(0);) | Cellular Metabolism | 8.33 | 0.06 | SEPT5,MZF1 | 5.04 | 0.00012 | 0.03859 |
| GO:0045921 | [positive regulation of exocytosis](javascript:void(0);) | Cellular Metabolism | 8.33 | 0.04 | SEPT5,MZF1 | 5.41 | 0.00006 | 0.03245 |
| GO:0010574 | [regulation of vascular endothelial growth factor production](javascript:void(0);) | Circulation | 8.33 | 0.03 | GATA4,MZF1 | 5.66 | 0.00004 | 0.02956 |
| GO:0010575 | [positive regulation vascular endothelial growth factor production](javascript:void(0);) | Circulation | 8.33 | 0.03 | GATA4,MZF1 | 5.81 | 0.00003 | 0.02956 |
| GO:0017157 | regulation of exocytosis | Cellular metabolism | 8.33 | 0.12 | SEPT5,MZF1 | 4.31 | 0.00045 | 0.04700 |
| GO:0019867 | [membrane](javascript:void(0);) protein | Cellular component | 12.5 | 0.44 | MZF1, RETSAT, MAVS | 3.48 | 0.00017 | 0.03973 |
| GO:0003916 | [topological transformation of dsDNA](javascript:void(0);) | Molecular function | 8.33 | 0.1 | TOP1,MZF1 | 4.51 | 0.00031 | 0.04388 |
| GO:0004437 | [phosphotase activity](javascript:void(0);) | Molecular function | 8.33 | 0.11 | PTPMT1,MZF1 | 4.43 | 0.00036 | 0.04388 |
| GO:0019905 | [Interacting selectively and non-covalently with a syntaxin](javascript:void(0);) | Molecular function | 8.33 | 0.11 | SEPT5,MZF1 | 4.43 | 0.00036 | 0.04388 |

Figure S1: a) FCA of resident populations on their own using neutral markers and b) the same for transient populations. SR= dark blue, AR= purple, RUS= brown, BS= black, AT= green, CT= orange.

a)


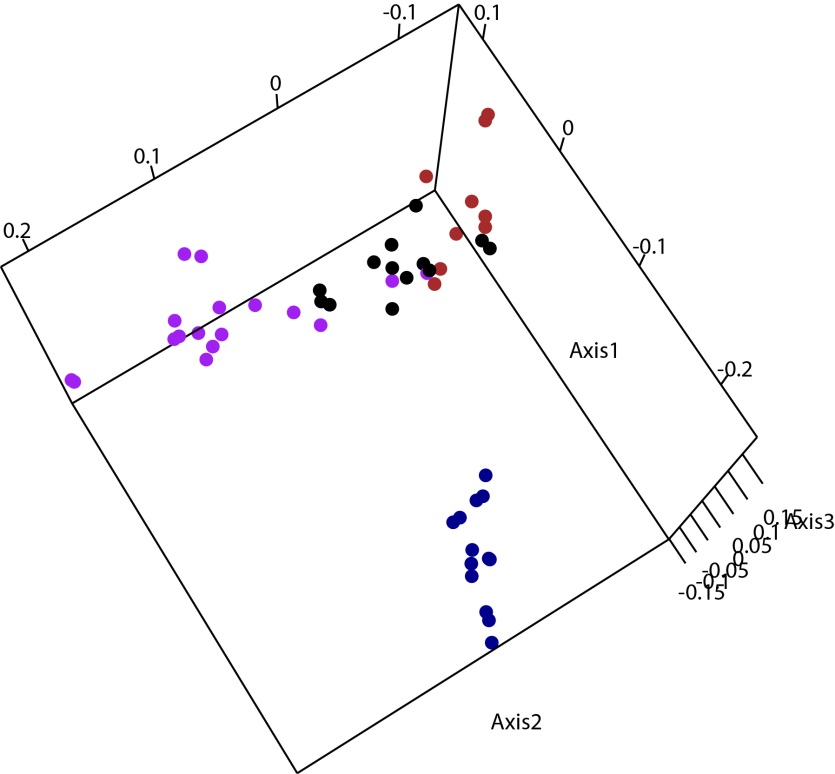


b)


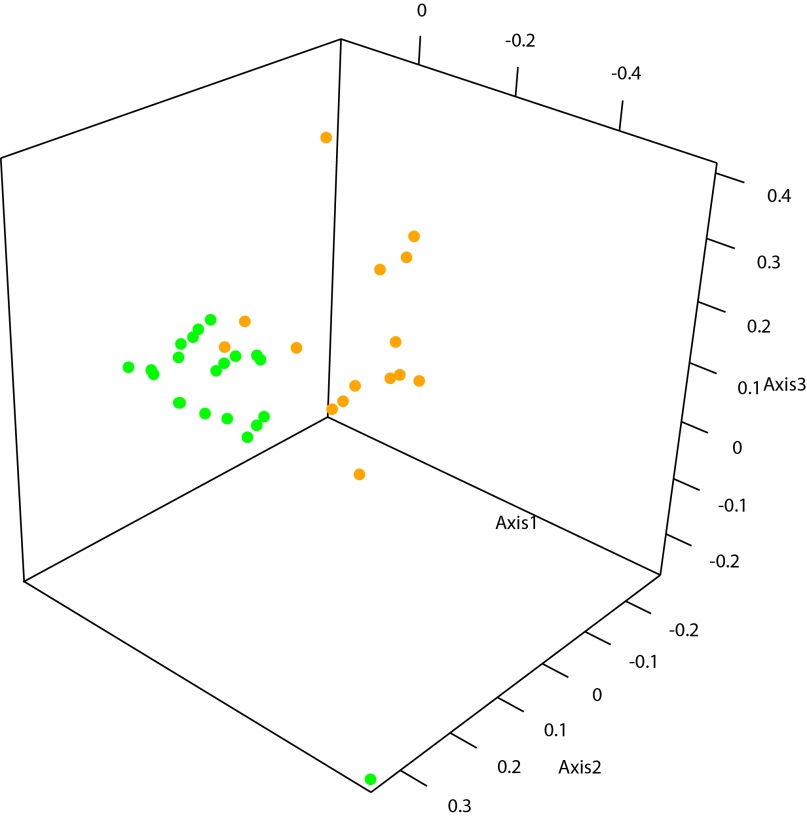


Figure S2: Pairwise comparisons of the allele frequency spectrum of the observed data (top row) versus the model (second row) and plots of the residuals (third and fourth rows) from ∂a∂i for the MI, AT, OS analysis.


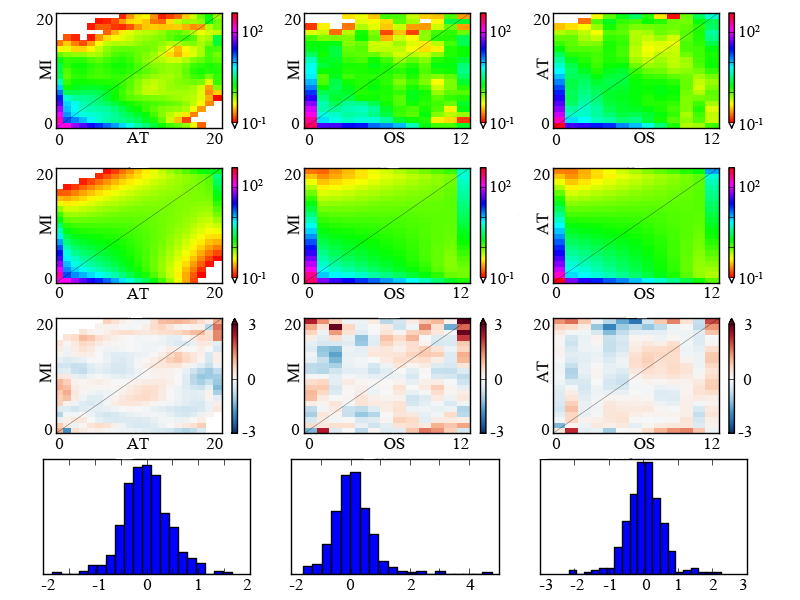


Figure S3: Pairwise comparisons of the allele frequency spectrum of the observed data (top row) versus the model (second row) and plots of the residuals (third and fourth rows) from ∂a∂i for the AT, OS, AR analysis.


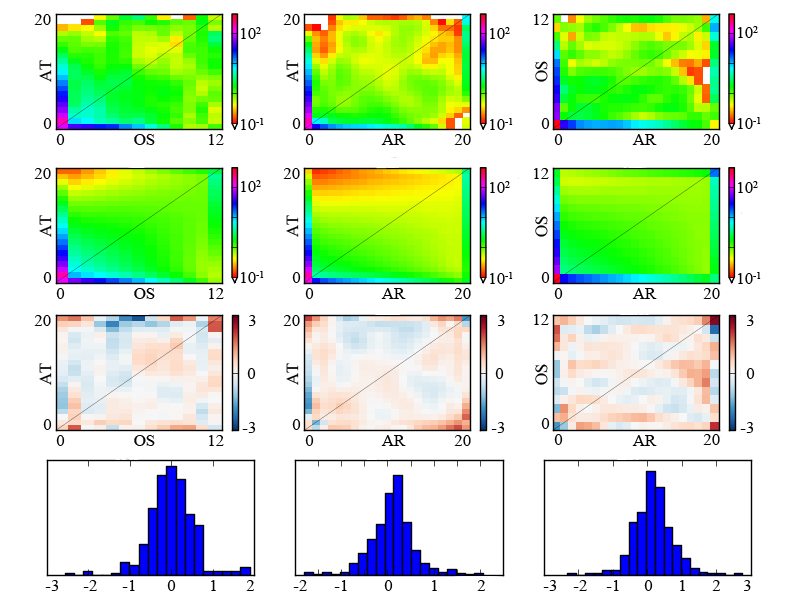


Figure S4: Individual FCA plot using the 33 SNPs that were fixed for a single allele in at least one *a priori* population.


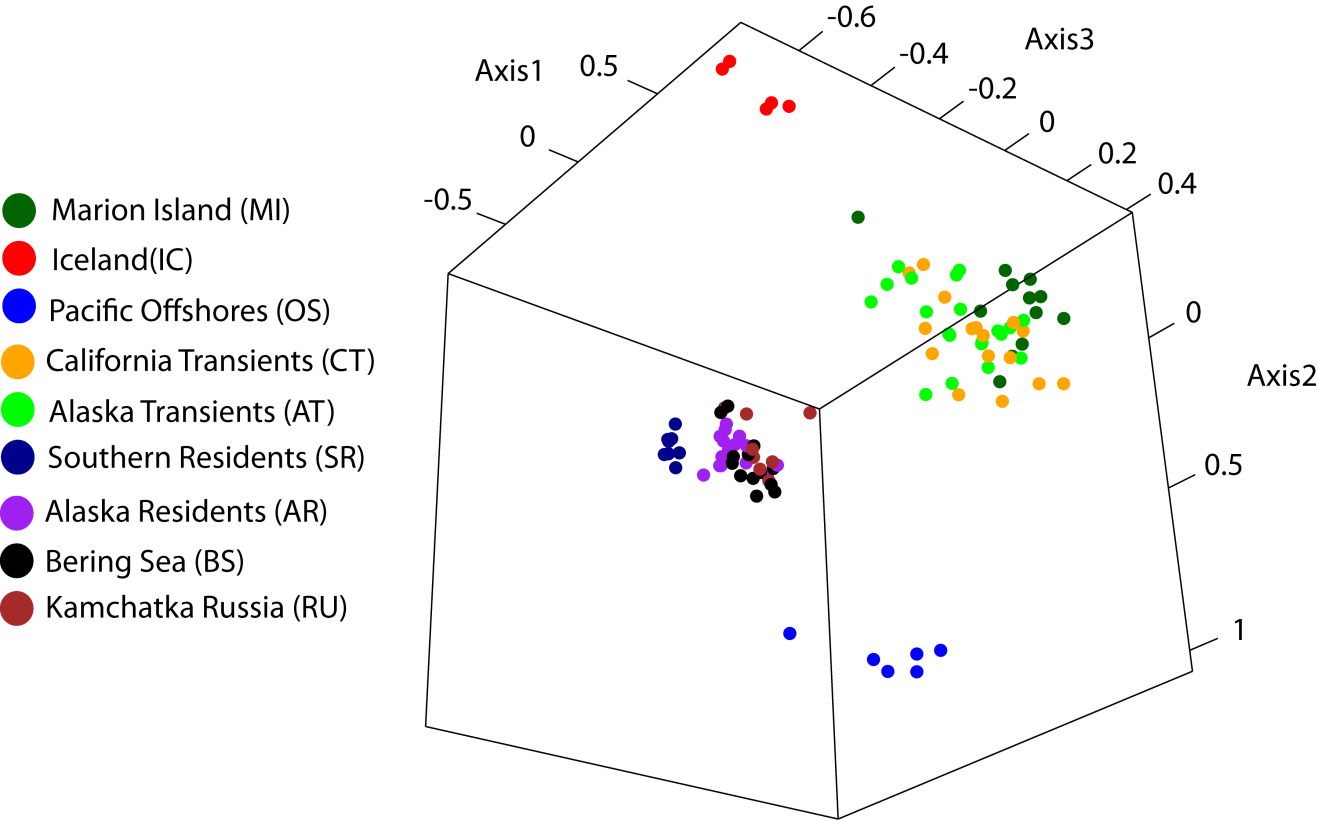

Supplement: Table S1 — Number of samples used in this study by predefined population. Table S2 Demographic estimates from ∂a∂i for the MI/AT/OS analysis. Table S3 Demographic estimates from ∂a∂i for the AT/OS/AR analysis. Table S4 Model comparisons for order of population splitting for the MI, AT, OS analysis. Table S5 Model comparisons for order of population splitting for the AT, OS, AR analysis. Table S6 Probability that observed fixed differences between population pairs were significant (bold and italics). Table S7 Genes listed by putative functions based on GO terms linked to strong outlier SNPs for positive selection (genes in bold show significant over-representation compared to reference GO terms; see Table S9). Table S8 Genes listed by putative functions based on GO terms linked to SNPs associated with fixed differences among putative populations (genes in bold show significant over-representation compared to reference GO terms; see Table S9). Table S9 List of significantly enriched GO terms as compared to the proportions in the human reference genome. Fig. S1 (a) FCA of resident populations on their own using neutral markers and (b) the same for transient populations. Fig. S2 Pairwise comparisons of the allele frequency spectrum of the observed data (top row) versus the model (second row) and plots of the residuals (third and fourth rows) from ∂a∂i for the MI, AT, OS analysis. Fig. S3 Pairwise comparisons of the allele frequency spectrum of the observed data (top row) versus the model (second row) and plots of the residuals (third and fourth rows) from ∂a∂i for the AT, OS, AR analysis. Fig. S4 Individual FCA plot using the 33 SNPs that were fixed for a single allele in at least one a priori population. [file mec0023-5179-SD1.docx]
